# Decorin‐mediated inhibition of the migration of U87MG glioma cells involves activation of autophagy and suppression of TGF‐β signaling

**DOI:** 10.1002/2211-5463.12076

**Published:** 2016-05-31

**Authors:** Ting Yao, Chen‐guang Zhang, Ming‐tao Gong, Min Zhang, Lei Wang, Wei Ding

**Affiliations:** ^1^ Department of Medical Genetics and Developmental BiologyCapital Medical UniversityBeijingChina; ^2^ Department of Biochemistry and Molecular BiologyCapital Medical UniversityBeijingChina; ^3^ Beijing Key Laboratory for Researches in Cancer Invasion and MetastasisCancer Institute of Capital Medical UniversityBeijingChina; ^4^ Department of NeurosurgeryBeijing Tiantan HospitalChina; ^5^ Beijing Institute of Brain DisordersChina

**Keywords:** autophagy, decorin, glioma, migration, TGF‐β signal

## Abstract

Decorin (DCN) is a major member of the small leucine‐rich proteoglycan (SLRP) family that is critically involved in tumorigenesis and the development of metastasis of cancers, including glioma. Overexpression of DCN was indicated to suppress glioma cell growth. However, the role of DCN in the migration of glioma cells remain elusive. In this study, we found that treatment with exogenous DCN inhibited the adhesion and migration of U87MG glioma cells with down‐regulation of TGF‐β signaling. DCN also activated autophagy, as indicated by monodansylcadaverine (MDC) staining, increase in LC3 I/LC3 II conversion, and p62/SQSTM1 degradation in U87MG cells. The increased activity of autophagy was found to be connected to the inhibition on glioma cell migration. Knockdown of DCN expression or the disruption of autophagy with 3‐methyladenine (3‐MA) was able to reduce the suppression on cell adhesion and migration induced by DCN. When U87MG cells were treated with temozolomide (TMZ), induction of autophagy and up‐regulation of DCN were observed, accompanied by suppressed cell adhesion and migration. Transfection of siRNA targeting DCN attenuated the suppressive effect of TMZ on glioma cell migration and adhesion. Our results indicated that the migration of glioma cells was under the control of the active status of autophagy, with DCN serving as a key player, as well as an indicator of the outcome. Therefore, it is suggested that autophagy‐modulating reagents could be considered for the treatment of invasive glioma.

**Abbreviations**
3‐MA: 3‐methyladenine
ATGs: autophagic‐related genes
CGGA: Chinese Glioma Genome Atlas
CQ: chloroquine
CSF: cerebro‐spinal fluid
DCN: decorin
ECIS: electric cell substrate impedance sensing
EGFR: epidermal growth factor receptor
GBM: glioblastoma multiforme
GSCs: glioblastoma stem cells
HCQ: hydroxychloroquine
LC3: light chain 3
MAP1LC3: microtubule‐associated protein 1 light chain 3
MDC: monodansylcadaverine
MMP: matrix metalloproteinase
PAI: plasminogen activator inhibitor
SLRP: small leucine‐rich proteoglycan
TGF‐β: transforming growth factor‐β
TMZ: temozolomide
VEGFR2: vascular endothelial growth factor receptor 2

Decorin (DCN) is a classical member of the small leucine‐rich proteoglycan (SLRP) family, which displays versatile functions in a variety of cellular processes, including fibrogenesis, cell proliferation, apoptosis, and angiogenesis 1, 2, 3, 4. DCN is of great importance as a major component in both extracellular and intracellular matrix and plays critical roles in the pathogenesis of certain human diseases and disorders, especially in various types of cancers 5. DCN was reported to exert anticancer activities partially through its interplay with a range of growth factors and the corresponding receptors at the cell surface 6. For examples, down‐regulated EGFR expression and its tyrosine kinase activity can be triggered by the DCN binding to receptors, which resulted in the induction of p21 and cell cycle retardation 7. Such mechanism was suggested to contribute to the growth inhibition of a variety of tumors including breast, colon, prostate, and lung cancers 8, 9. Similarly, DCN also induces cancer cell growth arrest by suppressing the activities of Met and VEGFR2, which are important signals for neoplasia progression 10, 11. Direct interaction of DCN on TGF‐β ligands was also suggested, inducing attenuation of TGF‐β signaling under certain circumstances 3.

In addition to the extracellular mechanisms, recent reports have indicated that DCN was able to modulate cell behavior through intracellular mechanisms, particularly autophagy 12, 13, 14, which was recognized for much biological importance. Autophagy is a conserved and dynamic process in vast eukaryotic cells and is known to be tightly regulated by a group of autophagic‐related genes (ATGs). The process of autophagy involves the sequestration of cytoplasmic components, including organelles, into double‐membrane structures known as autophagosomes, which can fuse with the lysosomes for the supply and turnover of nutrients and organelles 11. During transformation, autophagy generally exerts an anticarcinogenic role by counteracting metabolic stresses. In established cancers, the cancer cells may, however, activate autophagy as a protective mechanism, especially when challenged under stresses from chemotherapies 15. Decorin‐induced autophagy was lately found in a Met‐PEG3 axis‐dependent manner that was described in endothelial cells 12, 13. The implication from this finding sparked wide interests in the study of tumors. Decorin‐suppressed breast carcinoma growth was observed, during which induced mitophagy was found with the requirement of mitostain 14. Nonetheless, the exact role of DCN‐induced autophagy for the suppression of tumor cells demands further investigation, especially in specific invasive cancers like glioma.

Glioma, especially glioblastoma multiforme (GBM), is characterized by the diffuse infiltrative growth into the surrounding brain parenchyma. Such tumors are prone to incomplete surgical resection and local recurrences, bringing in a great challenge for classical cancer therapy 16. The migration and invasion of glioma cells greatly depends on the activation status of TGF‐β signals. TGF‐β ligand elicits its signal transduction via heteromeric type I and II transmembrane serine/threonine kinase receptor complex and the downstream Smad proteins to regulate the expression of many target genes 17. A number of TGF‐β‐regulated genes are responsible for glioma progression, including matrix metalloproteinase (MMP) family members and vascular endothelial growth factor (VEGF) 18, 19. DCN had been reported to exert an inhibitory effect on cell migration in several types of nonglioma tumors 20, 21, 22, and was suggested to be involved in the inhibition of cell growth and proliferation in glioma partially through blunting TGF‐β signals both *in vitro* and *in vivo* 23, 24. Although possible inhibition of DCN on cell migration and invasion has been implied in glioma 25, 26, the precise function and the possible mechanisms has not yet been elucidated.

In this study, the effect of DCN on the migration of glioma cells was investigated. We found that both addition of DCN protein core or overexpressed DCN was able to drastically suppress the migration of glioma cells. During this process, autophagy was activated and TGF‐β signaling was substantially compromised.

## Results

### Decorin inhibited the adhesion and migration of glioma cells

**Figure 1:**
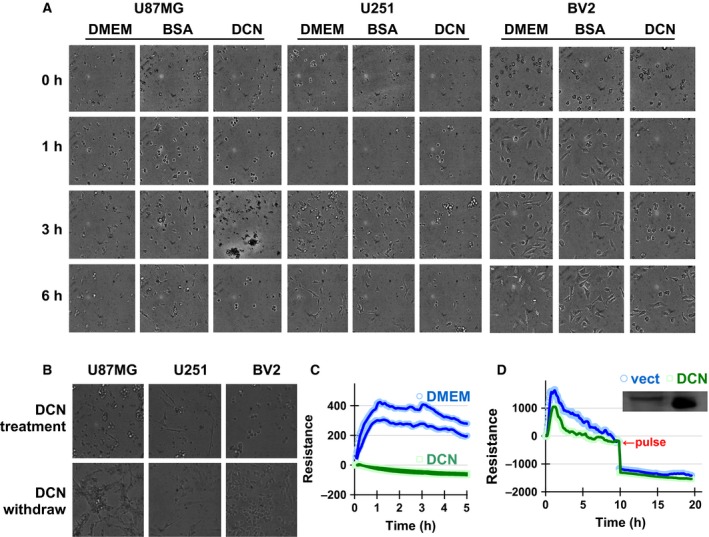
DCN inhibited the adhesion of glioma cells in culture. (A) U87MG, U251, BV2 cells were treated with 200 nm of recombinant human decorin protein core for extended periods. Cell adhesion statuses were observed under a microscope at different time points after seeding and compared to the medium and BSA controls. The images shown are representative of three independent experiments. (B) Effect of DCN withdrawn on cell adhesion through a fresh medium change at 24 h from continuous observation. The images shown are representative of three independent experiments. (C) U87MG cells were plated on the 8W1E ECIS chambers with or without additions of DCN protein core. The impedance was recorded. (D) The cells were transfected with a DCN overexpression plasmid prior to plating and traced for 20 h, during which a current pulse was applied at 5 h. Representative results from at least three times experiments were shown and compared with the vector transfection controls.

We initiated our study by adding DCN protein core into the medium of U87MG cells to observe if any morphologic changes might occur. We found the cells normally adhered onto the plates and finished adhesion by 6 h, which was not observed in 200 nm DCN‐treated cells. In U251 (human glioblastoma) and BV2 (murine microglia) cells, similar phenomena were observed (Fig. 1A). To exclude the possibility that the poor attachment of cells was due to cell toxicity or cell death, we removed DCN through a medium change and additional washing. We observed that the adherent ability of DCN‐treated cells was restored (Fig. 1B). This indicated that the reduced cell adherence was a consequence of DCN treatments and such an action of DCN appeared to be reversible. We then tried to examine the effect of DCN on cell adhesion using a more objective and dynamic measure. We deployed an electric cell‐substrate impedance sensing (ECIS) system for continuous tracing of the impedance. In DCN‐treated groups, the poor attachment of cell was indicated by the persistent low levels of time‐lapse measures (Fig. 1C, green line). In contrast, the impedance of the control cells sharply rose within 1 h and was maintained for 4 h or longer (Fig. 1C, blue). We further assayed the migration with ECIS in U87MG cells transfected with a decorin‐expressing plasmid. We observed that the migration of transfected cells was significantly reduced as shown by the attenuated recovery of the impedance as compared with that from the control samples (Fig. 1D).

### Decorin‐inhibited glioma cell migration associated with suppression of TGF‐β signaling

**Figure 2:**
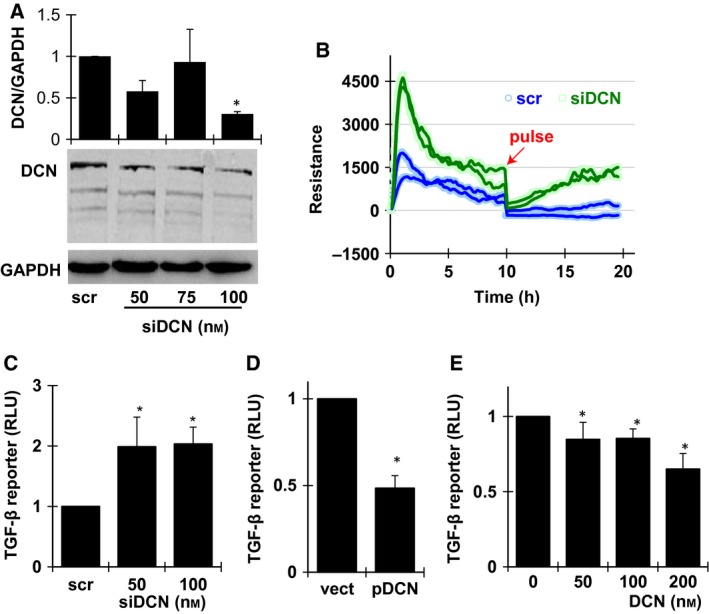
DCN inhibition on cell migration involved suppressed TGF‐β signals in U87MG cells. (A) Western blot for DCN knockdown efficiency at 48 h post transfection of different amounts of siDCN and quantification of the protein levels of DCN, respectively, from three independent experiments, data are shown as mean ± SEM. *P < 0.05. scr, scramble siRNA. (B) ECIS assays using 8W1E champers for cell migration with 100 nm siDCN transfection. The impedance was examined for 20 h and compared with the scramble control. (C) Cells of 10^4^ per well were seeded in 96‐well plates. The luciferase activities were determined at 48 h post transfection of 100 ng TGF‐β reporter plasmid together with 100 nm of scramble siRNA or siDCN. (D) U87MG cells were transfected with 80 ng TGF‐β reporter concomitantly with 80 ng control or DCN overexpression plasmid. Luciferase activity was examined at 48 h post transfection. (E) The cells were transfected with 100 ng TGF‐β reporter and exposed to different amounts of exogenous DCN protein core for 6 h prior to luciferase assays. All experiments were repeated at least three times and the data are expressed as mean ± SEM. *P < 0.05 versus control group (scramble siRNA, empty vector, or no DCN added groups for C, D, E panels, respectively).

We further confirm the suppressive effect of DCN on glioma cell migration by knocking down the expression of endogenous DCN with siRNA. To optimize the conditions for DCN RNA interference (RNAi) efficacy, we transfected the U87MG cells with different amounts of synthetic siDCN duplex and examined the protein level of DCN at 48 h post transfection. The dose of 100 nm was selected (Fig. 2A), where significant knockdown level and minimum toxicity was observed (data not shown). In Fig. 2B, reduced cellular DCN significantly increased the adhesion and migration of U87MG cells, as shown from the comparison between the siDCN groups (green lines) and the scramble siRNA controls (blue). As the regulation of cell migration often requires the activation of TGF‐β signal pathway, we employed the PAI promoter/luciferase reporter system to detect TGF‐β activities in U87MG cells. As shown in Fig. 2C, knockdown of DCN with 50 and 100 nm siDCN indeed significantly up‐regulated TGF‐β reporter activity, which was consistent with the ECIS measures for cell migration. Conversely, transfection with DCN overexpression plasmid suppressed TGF‐β signals, similar to the conditions when cells were treated with different amounts of exogenous DCN protein core (Fig. 2D,E). These results suggested that DCN‐mediated inhibition of glioma cell migration involved the suppression of TGF‐β signaling.

### Decorin‐induced autophagy in glioma cells

**Figure 3:**
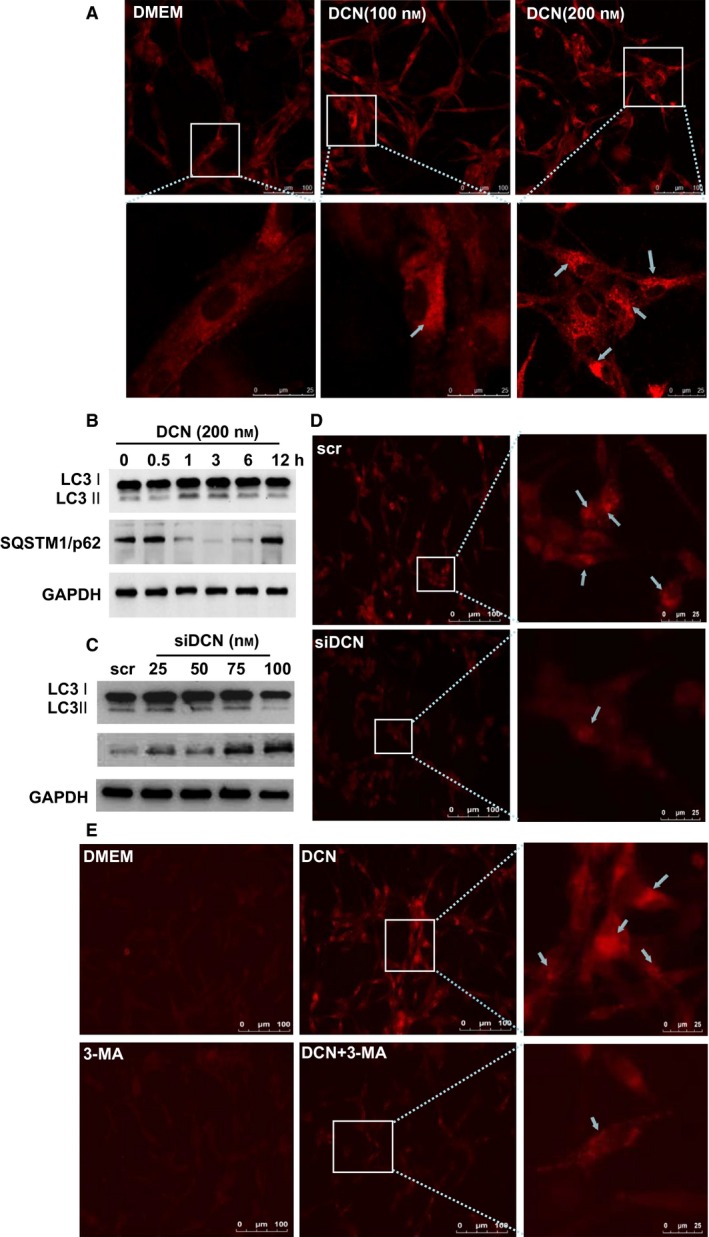
DCN‐activated autophagy in U87MG cells. (A) MDC staining of cytoplasmic autophagosomes (arrows) in U87MG cells treated with DCN for 6 h. The boxed regions were enlarged in corresponding lower panels. The images shown are representative of three independent experiments. (B) Western blot analyses of LC3 and p62/SQSTM1 in U87MG cells in 200 nm DCN at different time lapse. (C) Western blots of LC3 and p62/SQSTM1 in U87MG at 48 h after transfection with increasing doses of siDCN. (D) Confocal microscopy of U87MG cells transfected with 100 nm siDCN at 24 h. The images shown are representative of three independent experiments. (E) Confocal images of U87MG cells treated with 200 nm DCN for 6 h in the presence or absence of 1 mm 3‐MA. Arrows pointed to LC3 puncta. Nuclei were stained with DAPI. The images shown are representative of three independent experiments.

Decorin could induce autophagy in endothelial cells or breast cancer cells through VEGFR2 or Met receptor 12, 13, 14. We further investigated if DCN is able to activate autophagy in glioma cells. To this end, we first used the method of monodansylcadaverine (MDC) staining to visualize the autophagosome formation with confocal microscopy. DCN protein core treatments caused accumulation of autophagosome‐like structure in the cytoplasm of U87MG cells in a dose‐dependent manner (Fig. 3A). Meanwhile, the hallmarks of autophagy activation, the conversion of light chain 3‐I (LC3 I) to LC3‐II and the selective degradation of p62/SQSTM1 were detected in immunoblots, especially under conditions when 200 nm of DCN was applied for 3 h (Fig. 3B). On the contrary, as U87MG cells transfected with increasing doses of DCN siRNA, the conversion of LC3 I to LC3 II decreased, p62/SQSTM1 levels increased (Fig. 3C), suggesting that DCN may function in maintaining the basal autophagy level in glioma cells. Consistently, diminished accumulation of LC3 puncta was observed in U87MG cells transfected with siDCNs (Fig. 3D). We further used 3‐MA to pretreat the cells before adding DCN to the culture medium. Compared with the Dulbecco's modified Eagle's medium (DMEM) group, the induction of autophagy was significantly attenuated as evidence with reduced level of LC3 puncta. This indicated that DCN‐induced autophagy in glioma cells was canonically dependent on class III phosphoinositide 3‐kinase (Fig. 3E).

### Autophagy was involved in decorin‐induced inhibition on TGF‐β activity and cell migration in U87MG cells

**Figure 4:**
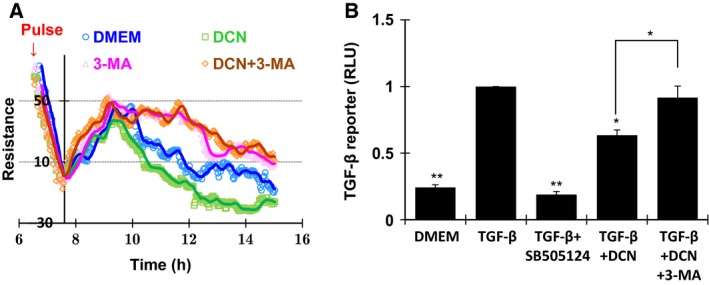
Autophagy was required for DCN‐induced suppression of migration and TGF‐β activity in U87MG cells. (A) Effect of DCN on U87MG cell migration was characterized in the absence or presence of 3‐MA by ECIS. (B) Luciferase assay of TGF‐β signal at 6 h of DCN treatment with or without 3‐MA in U87MG cells. Exogenous TGF‐β1 protein (20 ng·mL^−1^) or SB505124 (100 μm) was used as positive or negative controls. Data from three independent experiments were normalized and shown as Mean ± SEM (*P < 0.05; **P < 0.01).

To evaluate the role of autophagy in DCN‐induced migration inhibition and TGF‐β suppression in glioma cells, we treated U87MG cells with 3‐MA for ECIS assays. The results showed that 3‐MA treatment alone promoted cell migration as compared to the DMEM (Fig. 4A, pink and blue), suggesting that the cell migration process might require the participation of autophagy machinery. Interestingly, the postaddition of DCN in 3‐MA‐treated cells was not able to restore cell migration to a significant level, while higher impedance was observed over the 200‐nm DCN‐treated U87MG cells (Fig. 4A, brown and green). Accordingly, DCN treatments reduced TGF‐β activity as demonstrated by reporter assays, which was attenuated following the pretreatment with 3‐MA. Using SB505124, an inhibitor of TGF‐β signals, we were able to demonstrate that the effect of DCN was direct and specific (Fig. 4B). These results implied that the inhibitory effect of DCN on cell migration and TGF‐β signaling pathway was dependent on autophagy process.

### Decorin was necessary for autophagy induction, cell migration, and TGF‐β signaling inhibition in temozolomide‐treated glioma cells

**Figure 5:**
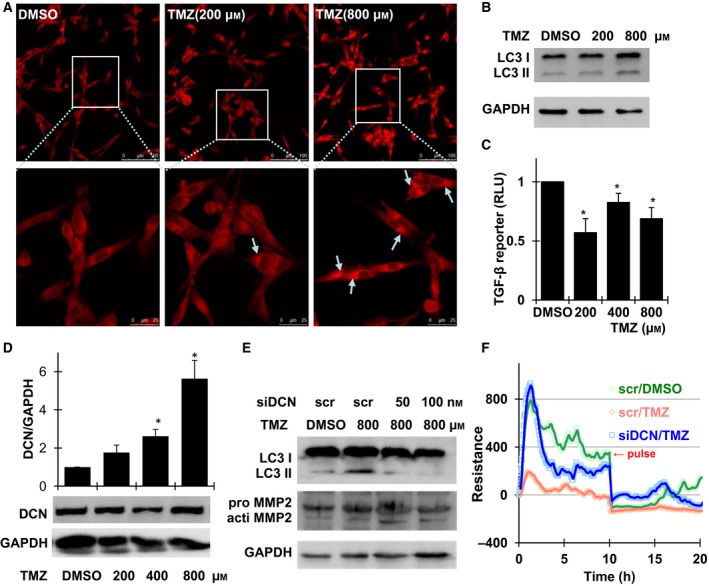
TMZ treatments inhibited glioma cell adhesion and migration through autophagy induction and suppression of TGF‐β signal. (A) MDC staining of U87MG cells treated with different dose of TMZ for 6 h. Cytoplasmic autophagosomes were indicated by arrows. The images shown are representative of three independent experiments. (B) Western blot of LC3 I and II in U87MG cells exposed to different concentrations of TMZ for 6 h. (C) Luciferase assay for TGF‐β activities in TMZ‐treated U87MG cells. (D) Western blot of DCN expression upon TMZ treatments and quantification of the protein levels of DCN, respectively, from three independent experiments; data are shown as mean ± SEM. *P < 0.05. (E) U87MG cells were transfected with siDCN or a scramble control for 24 h, Western blotting was performed following 800 μm TMZ treatment for 6 h for LC3 and MMP2. (F) The adhesion and migration of U87MG cells was assayed using an ECIS system following 100 nm siDCN transfection of 24 h and continuous exposure to 800 μm TMZ.

Temozolomide (TMZ) is a most frequently used chemotherapeutic drug for glioma treatments. TMZ induces massive DNA damages and triggers cell apoptosis of glioma cells 27. TMZ was reported to also activate autophagy in U87MG cells 28. We observed a similar effect as indicated by MDC staining (Fig. 5A). The enhancement of autophagy was further confirmed by the increase in converted LC3 II levels in TMZ‐treated U87MG cells, which was obviously observed at a higher level of TMZ (Fig. 5B). Meanwhile, the TGF‐β activity and cell migration was found decreased as evidence by luciferase reporter and ECIS assay in glioma cells following TMZ exposure, respectively (Fig. 5C,F brown and green). An interesting finding was that TMZ was able to dose‐dependently induce robust overexpression of endogenous DCN (Fig. 5D). Suppressing of the TMZ‐induced DCN by siDCN transfection significantly compromised the inhibition of cell migration from TMZ treatment (Fig. 5F, blue and brown), which was correlated with the level changes in MMP2 activation (Fig. 5E). Meanwhile, knockdown of DCN also reduced the activation of autophagy induced by TMZ (Fig. 5E). Taken together, these data suggested that DCN inhibition on glioma migration and TGF‐β signaling were closely related or probably through autophagy to mediate the effect of TMZ.

## Discussion

Decorin was well known to exert suppressive effects on the growth of tumors, and its effect on the migration was investigated in several cancer types 29, 30, 31, 32, but not yet for gliomas prior to our current study. As the most progressive malignancy caused by invasive propagation occurs in GBM, we chose a representative cell line of U87MG as our primary experiment model. We found that either soluble DCN protein core or overexpression of DCN exhibited a potent antimigration effect in glioma cells (Figs 1D and 4A). It needs to be pointed out that DCN inhibition on cell migration can be complicated at the tissue level in patients, whereas DCN may function at both intracellular and extracellular levels. The fact that DCN expression could be boosted by TMZ treatments (Fig. 5D,F) implied a sophisticated DCN‐mediated outcome for glioma metastasis in real patients undergoing chemotherapies. From the laboratory experiment, we found that knockdown of DCN levels partially damaged the TMZ suppression on cell migration (Fig. 5F). This could be related to the decreased basal cellular DCN level, which was possibly required for maintaining normal migratory events in U87MG cells (Fig. 2B). Besides TMZ, other factors like adiponectin have been shown to induce DCN expression 33, which might be involved its antiglioma effects 34. These findings suggested that DCN treatments was a causal and direct stimuli to induce migration inhibitory effect, which also probably existed as a common intermediate mechanism underlying the suppressive processes on glioma cell migration under different circumstances.

TGF‐β signaling plays a pivotal role in regulating the migration of glioma cells. It was not a surprise when we found that DCN‐mediated suppression of glioma cell migration was correlated with the inhibition of TGF‐β activity (Fig. 2C,D,E). Previous discovery of DCN actions on migration was widely believed as the consequence of extracellular sequestration of TGF‐β ligands 3. Recently, it was recognized that DCN‐induced autophagy and autophagy was known to modulate TGF‐β signaling and cell migration 35. This prompted us to re‐evaluate the effect of DCN on TGF‐β and cell migration from the perspective of autophagy. We found that soluble decorin protein core potently induced autophagy in U87MG glioma cells, as evidenced by both the accumulation of LC3 and the degradation of p62 (Fig. 3B and C). The blockage of autophagy induction with 3‐MA significantly reduced the suppressive effect of DCN on TGF‐β signaling and cell migration (Fig. 4A,B). Further detailed analysis indicated that DCN‐induced autophagy occurred as early as 1 h (Fig. 3C), prior to the decline of TGF‐β reporter activity (Fig. 2E). It suggested that the role of autophagy in the regulation of cell migration possibly mediated by the inhibition of TGF‐β signaling. Previous reports concerning the role of autophagy in glioma cell migration appeared to be controversial 36, 37, 38. Our results implicated that the regulation of autophagy on cell migration was context dependent, differing with cells types or stimuli. The mechanism underlying the autophagy induction by DCN in glioma cells was still unknown. Given the expression of VEGFR‐2 and Met in U87MG cells 39, 40, it could not preclude the possibility that DCN induces autophagy through either or even both receptors. Furthermore, how autophagy modulates the TGF‐β signaling in this model also needs to be further investigated.

Autophagy played a dual role in TMZ‐treated glioma cells. Inhibition of autophagy by 3‐MA at the early stages reduced TMZ efficacy for tumor suppression; while the blockage of later autophagy stages with bafilomycin enhanced TMZ effects 38, 41. Induction of autophagy by TMZ in U87MG cells linked to the suppression of TGF‐β signaling (Fig. 5A,B,C), and such an effect appeared to be dependent upon the existence of DCN (Fig. 5E). An immediate possible explanation would be that DCN was a required player in TMZ‐induced autophagy, which in turn suppressed the glioma cell migration through TGF‐β signaling. Further studies are needed to elaborate the interrelations among DCN, autophagy, and TMZ, where autophagy inhibitors like Chloroquine (CQ) and its derivative HCQ could be considered, as these compounds are now being tested for trials in glioma treatments 42.

In seeking the clinical relevance of the current findings, data from the Human Protein Atlas 43 (HPA, www.proteinatlas.org) were retrieved and used for analyses. An increased level of DCN in glioma patients was found as compared with paracancerous tissues (Fig. S2A). Meanwhile, the normalized microarray data from the Chinese Glioma Genome Atlas (CGGA, www.cgga.org.cn) were evaluated for the DCN mRNA. A similar increased trend was shown as the WHO classification grade of glioma proceeds, where grade IV reached the highest level (Fig. S2B). Patients with higher DCN expression was indicated for decreased survival probabilities from the CGGA data (Fig. S2C). We selectively examined the cerebro‐spinal fluid (CSF) level of DCN from glioma patients or other non‐neoplastic disorders with pooled samples, the results seemed in support of the conclusion from microarray analyses (Fig. S2D). Taken together, the increased DCN levels in glioma tissues may turn out not to be a factor participating in glioma progression as initially suspected, rather it implies that decorin acts as a suppressor for both cancer growth and metastasis. However, such an inhibitory effect of DCN on cancer progression is likely to require a relative healthy autophagy status or an appropriate autophagy capacity in tumor cells. The theory discussed here may be applied to other tumor types, as similar DCN expression profiles were found also in colon cancers 44, where increased DCN correlated with suppressed tumor growth. The mechanisms underlying the up‐regulation of decorin in glioma needs further investigation.

In conclusion, we discovered that decorin exhibited an antimigration effect in U87MG glioma cells with the induction of autophagy and consequent inhibition of TGF‐β signaling. This finding not only provided a possible mechanism to explain the effect of decorin on glioma cell migration but also suggested the potential of DCN to be employed as an agent for evaluating glioma treatments. Meanwhile, our investigation also emphasized the importance for considering the role of autophagy activation with its implications for the therapeutic significance in cancers, where currently several autophagy inhibitors have been considered for clinical trials, including gliomas.

## Materials and methods

### Cell culture, transfection, and cerebro‐spinal fluids

The U87MG cell line was purchased from the cell bank at Peking Union Medical University and maintained in high glucose DMEM supplemented with 10% FBS (Hyclone, Beijing, China). Transfection of plasmids or siRNA was conducted using Fugene HD reagent (Roche, Penzberg, Germany) or Lipofectamine RNAiMAX reagent (Invitrogen, New York, NY, USA)/OPTI‐MEM (Gibco, New York, NY, USA), respectively, following the manufacturer's instructions.

The cerebro‐spinal fluids (CSFs) were provided by Beijing Shi Ji tan hospital. All human materials including CSF have been approved by the institutional review board, and each patient provided a written informed consent.

### Plasmids, chemical reagents, and antibodies

The GFP‐p62 plasmid was kindly provided by T. Johansen (University of Tromsø, Tromsø, Norway). The GFP‐LC3 plasmid was a generous gift from C. Ying‐Yu (Peking University, Health Science Center). The decorin overexpression plasmid and the human plasminogen activator inhibitor‐1 (PAI‐1) promoter/luciferase reporter plasmid that is responsive to TGF‐β were from L. Shen‐tao (Department of Immunology, Capital Medical University). The reagents of 3‐methyladenine (3‐MA, Cat. No. M9218‐100MG), MG132 (Cat. No. C2211‐5MG), rapamycine (Cat. No. 37094), SB505124 (Cat. No. S4696), chloroquine (Cat. No. 6628), temozolomide (Cat. No. T2577‐25MG), recombinant human decorin protein core (rhDecorin, rhDCN, Cat. No. D8428), Monodansylcadaverine (MDC, Cat. No. D4008), ELISA Kit for decorin (Cat. No. RAB0140) were purchased from Sigma‐Aldrich (St. Louis, MO, USA). Luciferase assay kit (Cat. No. E1500) was purchased from Promega Corporation (Madison, WI, USA). Antibody of mouse anti‐decorin (Cat. No. AF1060) and recombinant human TGF‐β (rhTGF‐β) protein (Cat. No. 240‐B) were purchased from R&D Corporation (Minneapolis, CA, USA). The use of antibodies of rabbit anti‐p62 (Cat. No. P0067; Sigma‐Aldrich), HRP‐conjugated mouse anti‐GAPDH (Cat. No. KC‐5G5; Kangchen‐BioTech, Shanghai, China), rabbit anti‐LC3 (Cat. No. 1274), or ATG7 (Cat. No. 2631) (Cell signaling technology, Danvers, MA, USA) strictly followed the manufacturer's recommendations.

### RNA interference

Decorin‐targeting siRNA of 5′‐CGACUUUAUCUGUCCAAGAAU‐3′ was used to transfect U87MG cells in 6‐well plates. A scrambled siRNA (Augct, Beijing, China) in 100 nm was used as the control. Total proteins or RNA were extracted at 48 h post transfection. Optimized conditions for siRNA inhibition of the targeted genes were tested and the knockdown efficiency was determined by both western blot and quantitative PCR (qPCR).

### Immunofluorescence microscopy

Cells were grown on coverslips and treated with DCN protein core for 6 h, and then fixed in 4% paraformaldehyde (Cat. No. 16005; Sigma‐Aldrich) for 15 min at room temperature. After rinsing with PBS for three times (5 min for each time), the cells were permeablized with 0.5% Triton X‐100 (Cat. No. AR‐0341; DingGuo ChangSheng Biotechnology, Beijing, China) for 15 min and blocked with 1.5% Bovine Serum Albumin (Cat. No. 0332, BSA; Amresco, Solon, OH, USA) in PBS for 1 h, then incubated with primary antibodies (1 : 100) overnight at 4 °C and appropriate secondary antibodies (1 : 200, goat anti‐(mouse IgG) Alexa Fluro 488 and/or goat anti‐(rabbit IgG) Alexa Fluro 594; Life, Carlsbad, CA, USA). Confocal microscopy observation was performed with a Leica TCS SP5 MP system. Mounting medium with DAPI (4′,6‐diamidino‐2‐phenylindole, Cat. No. ZLI‐9557; ZhongShan JinQiao, Beijing, China) was used in fluorescent microscopy for the staining nuclei in fixed cells.

### Western blot

Cells in 6‐well plates were lysed in 80–100 μL of modified RIPA buffer (Thermo, Rockford, IL, USA) containing the full cocktail of protease inhibitors (Thermo). Protein concentrations were determined with a bicinchoninic acid protein assay kit (Novagen, San Diego, CA, USA). The amounts of 15 μg of protein per lane were loaded to 10 or 15% SDS/PAGE. After being transferred onto PVDF filters (Polyvinylidene Fluoride Membrane, Millipore, Billerica, MA, USA) and blocked with blocking buffer [TBST containing 1.5% (m/v) BSA] for 1 h, the filters were blotted with primary antibodies over night at 4 °C. After washing with TBST for three times, the membrane was incubated with secondary antibodies tagged with Horseradish Peroxidase (HRP) (Signalway Antibody LLC, College Park, MD, USA) for 1 h at room temperature, then visualized for bands with an enhanced chemiluminescence (ECL) (Millipore) detection system.

### RNA isolation, reverse transcription, and quantitative real‐time PCR

Total cellular RNA was extracted using 1 mL of TRIzol reagent (Invitrogen) per well in 6‐well plates. RNA in 400 ng amounts were used for cDNA synthesis with PrimeScript^™^ RT reagent Kit (Cat. No. RR037A; TaKaRa, DaLian, China) according to the manufacturer's standard protocol. Samples were subjected to a CFX Connect^™^ Real‐Time PCR Detection System (BioRad, Hercules, CA, USA) for analyses in triplicate. Endogenous housekeeping gene, beta‐actin (ACTB) or GAPDH were used as normalizing controls and amplified, using the SYBR^®^ Premix DimerEraser^™^ (TaKaRa). The cycle number (Ct) was calculated and the fold changes of gene expression were determined using the double ∆Ct (2^−∆∆CT^) method.

### Cell viability analysis

A CellTiter‐96 Aqueous‐One Solution Cell Proliferation (3‐(4,5‐dimethyl‐2‐thiazolyl)‐2,5‐diphenyl‐2‐H‐tetrazolium bromide, MTT) Assay Kit (Cat. No. G3580; Promega) was used. Cells were seeded in 96‐well plates at 0.5–1 × 10^4^ cells per well for 24 h, and then treated for conditions according to the experimental design. The MTT reagent was added into the culture medium and maintained for 30 min at 37 °C. The plates were subjected to measurements of the absorption at 490 nm with The ELx808^™^ Absorbance Microplate Reader (BioTek, Winooski, VT, USA).

### Electric cell substrate impedance sensing

The adhesion and migration of U87MG cells were determined with the electric cell substrate impedance sensing (ECIS) system (ECIS Ztheta; Applied BioPhysics, Troy, NY, USA) as described in 45, where the principal was delineated in Fig. S1. U87MG cells (native or pretransfected for 12 h with siDCN or decorin‐expressing plasmid) were seeded in 8‐well chambers (8W1E PET; Applied BioPhysics) and examined the impedance dynamically. To form the wounded area in cell migration assay, with adhered cells, a current pulse (3000 μA, 60 000 Hz for 40 s) was applied at the central area to induce controlled lesion area. The recovery of electrical impedance was monitored and recorded during the healing process from cell migration.

### Monodansylcadaverine staining

Cells were seeded on cover slips overnight followed by treatment with different doses of DCN protein core for 6 h and autophagosome‐like vacuoles were labeled with MDC by incubating the cells with 20 μm MDC in DMEM at 37 °C for 1 h. After incubation, cells were washed with PBS and fixed with a 4% paraformaldehyde for 20 min. Cells were examined under a Leica TCS SP5 MP system.

### Luciferase assay

U87MG cells were transfected with the PAI promoter/luciferase reporter in 96‐well plates. The transfected cells following different treatments were lysed, and mixed with the luciferase assay reagent (Cat. No. E1501; Promega). The luciferase activities were detected by a luminometer (Lumat LB 9507; Berthold Technologies, Stuttgart, Germany).

### Statistical analysis

Data were analyzed by one‐way anova, followed by the *post hoc* least significant difference test using spss 19 software (International Business Machines Corporation, Armonk, NY, USA). Results are presented as mean ± SEM, and statistical differences were determined as *P* < 0.05.

## Author contributions

TY did most of the experiments and acquired the data; C‐gZ analyzed the data and provided critical revision of the manuscript for important intellectual content; M‐tG and MZ offered material support and provided technical support; WD proposed the study design, supervised the study, and drafted the manuscript.

## Supporting information


**Fig. S1.** Schematic overview upon the working principle of an ECIS system. Cells were grown on the bottom of the cell culture chambers with previously deposited gold film electrodes. A separate counter electrode was planted to complete the measuring circuit. Adhesion of the seeded cells in the chamber resulted in the growing impedance traced by the system. An electric pulse could be used to create a ‘wound’, where the cells close to the electrode were bladed from cell death. During continued culture, survived cells around the electrode migrated to the ‘wounded area’ and recovered the dropped impedance measures. The ECIS system can be used to assay both cell adhesion and migration. The impedance recording at the post‐pulse phase will describe the migratory behavior of the cells.

**Fig. S2.** Over‐expression of decorin was frequently observed in glioma patients (A) Immunohistochemistry results from the Human Protein Atlas database (www.proteinatlas.org) indicated that the staining of decorin in glioma patients (http://www.proteinatlas.org/ENSG00000011465-DCN/cancer/tissue/glioma, available from v13.proteinatlas.org) was more intense as compared with non‐glioma samples (http://www.proteinatlas.org/ENSG00000011465-DCN/tissue/cerebral+cortex, available from v13.proteinatlas.org). (B) The normalized microarray data of DCN expression from CGGA documented 225 glioma patients. Increased decorin levels were correlated with disease progression with the highest observation in WHO grade IV glioblastoma. (C) The Kaplan‐Meier survival analysis based on the clinical data from CGGA patients, where high level (above the median value) of DCN expression indicated shorter survival expectancy. (D) The level of decorin increased in CSF of glioma patients determined by ELISA.
