# Supplementary figures and images for: Decorin‐mediated inhibition of the migration of U87MG glioma cells involves activation of autophagy and suppression of TGF‐β signaling
Source: FEBS Open Bio. 2016 May 31;6(7):707–19. doi: 10.1002/2211-5463.12076 (PMC4932450; doi:10.1002/2211-5463.12076)

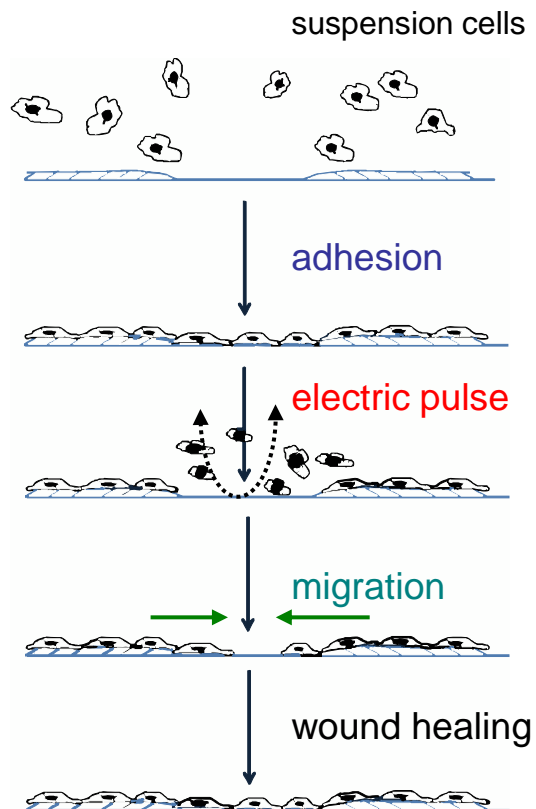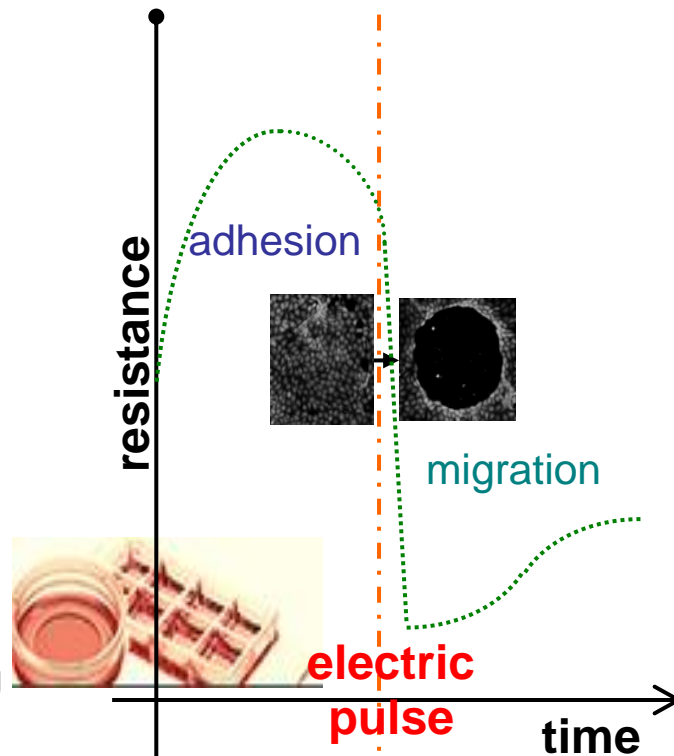

Supplement: Supplementary file 1 — Fig. S1. Schematic overview upon the working principle of an ECIS system. Cells were grown on the bottom of the cell culture chambers with previously deposited gold film electrodes. A separate counter electrode was planted to complete the measuring circuit. Adhesion of the seeded cells in the chamber resulted in the growing impedance traced by the system. An electric pulse could be used to create a ‘wound’, where the cells close to the electrode were bladed from cell death. During continued culture, survived cells around the electrode migrated to the ‘wounded area’ and recovered the dropped impedance measures. The ECIS system can be used to assay both cell adhesion and migration. The impedance recording at the post‐pulse phase will describe the migratory behavior of the cells. [file FEB4-6-707-s001.pdf]

**A**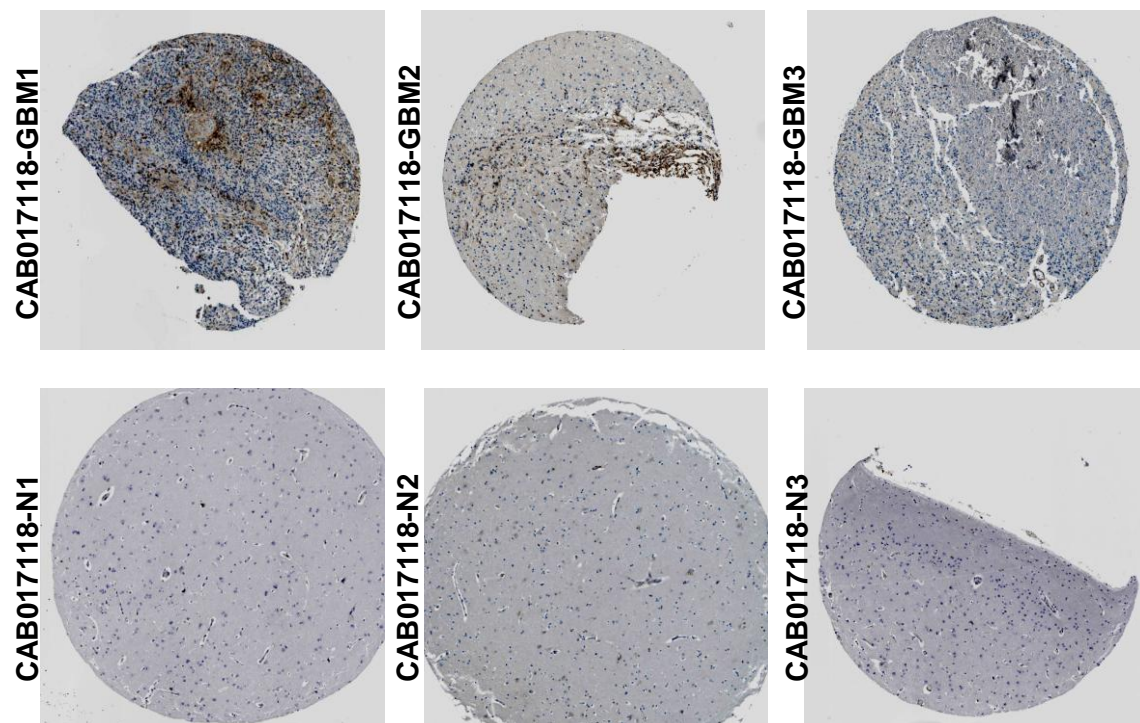**B**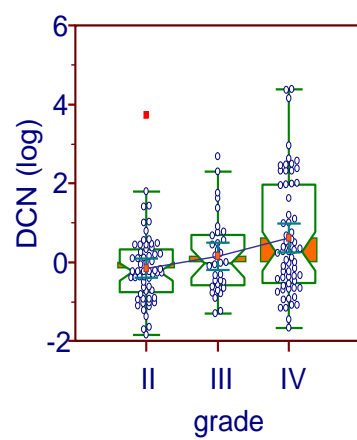**C**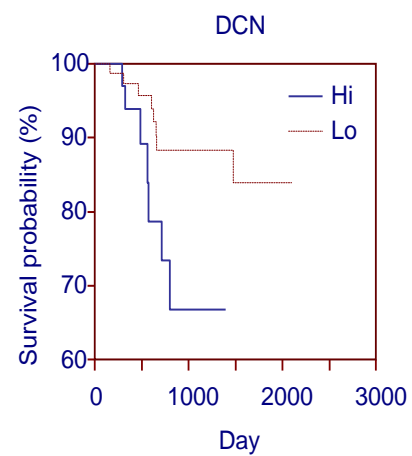**D**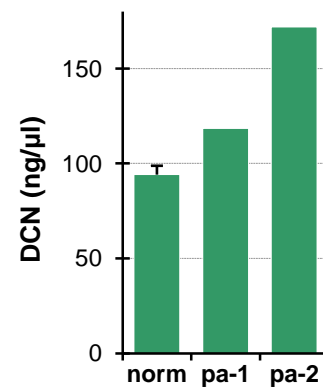

Supplement: Supplementary file 2 — Fig. S2. Over‐expression of decorin was frequently observed in glioma patients (A) Immunohistochemistry results from the Human Protein Atlas database (www.proteinatlas.org) indicated that the staining of decorin in glioma patients (http://www.proteinatlas.org/ENSG00000011465-DCN/cancer/tissue/glioma, available from v13.proteinatlas.org) was more intense as compared with non‐glioma samples (http://www.proteinatlas.org/ENSG00000011465-DCN/tissue/cerebral+cortex, available from v13.proteinatlas.org). (B) The normalized microarray data of DCN expression from CGGA documented 225 glioma patients. Increased decorin levels were correlated with disease progression with the highest observation in WHO grade IV glioblastoma. (C) The Kaplan‐Meier survival analysis based on the clinical data from CGGA patients, where high level (above the median value) of DCN expression indicated shorter survival expectancy. (D) The level of decorin increased in CSF of glioma patients determined by ELISA. [file FEB4-6-707-s002.pdf]
